# Arrested diversification? The phylogenetic distribution of poorly-diversifying lineages

**DOI:** 10.1038/s44185-022-00004-0

**Published:** 2022-12-22

**Authors:** Fernanda S. Caron, Marcio R. Pie

**Affiliations:** 1 Departamento de Zoologia, Universidade Federal do Paraná, C.P 19020, Curitiba PR, 81531-990 Brazil; 2 Biology Department, Edge Hill University, Ormskirk, Lancashire United Kingdom

**Keywords:** Evolutionary ecology, Macroecology

## Abstract

Rapidly-diversifying lineages have been a major focus of modern evolutionary biology, with many hypotheses seeking to explain how they contribute to the uneven distribution of species in space and among taxa. However, an alternative view that is rarely explored is that some lineages evolve to become depauperate clades and show disproportionately low diversification, in a phenomenon we here call arrested diversification. In this study, we analyse several large-scale datasets including amphibian, squamate, mammal, and seed plant species to assess the extent to which poorly-diversifying lineages show distinct phylogenetic and spatial distributions in relation to other lineages. We found significant evidence that clades with low diversification rates tend to be more phylogenetically overdispersed than expected and show more idiosyncratic spatial distributions. These results suggest that arrested diversification is a real phenomenon that might play an important (yet largely overlooked) role in explaining asymmetries in the distribution of species across lineages.

## Introduction

Biodiversity on Earth varies widely both in space and among taxa. For example, arthropods encompass most of the species diversity across all animal phyla, with over 1 million species^1^. Likewise, angiosperms include most of the known extant vascular plants, with over 295,000 described species^2^. However, even within these hyperdiverse clades, species diversity is still uneven, with a relatively small number of taxa accounting for most of the known diversity—such as the case of insects within arthropods^1^, and hymenopterans within insects^3^.

Of all current explanations for this unevenness in the distribution of species richness among lineages, possibly the most commonly invoked is the phenomenon of adaptive radiation, which is characterized by the rapid diversification of several species from a single common ancestor as a result of adaptation to distinct ecological niches^4–6^. Many instances of adaptive radiations, such as Darwin’s finches on the Galápagos Islands, the African Rift Lake cichlids, and the Hawaiian silverswords^4,7^ had a profound influence in shaping current evolutionary theory^5^. The main driver of adaptive radiation is thought to be ecological opportunity^8^, which becomes available for particular clades, such as after the origin of a key innovation^9,10^, the colonization of a new habitat^11^, or the extinction of an ecologically dominant group^12,13^.

Despite the importance of the concept of adaptive radiation has in evolutionary biology, there is another potential explanation for variation in species diversity across clades that is rarely considered. This explanation suggests that the unevenness of species richness might arise because some clades actually decrease their diversification rates, remaining poorly diverse^14,15^. Potential examples of this phenomenon include the lineages commonly known as living fossils, which are characterized by their morphological stasis and limited diversification, resulting in low diversity^16^. Here, we compiled a large dataset of plant and animal clades to assess the phylogenetic and geographical distributions of poorly-diversifying lineages. We demonstrate that these lineages tend to be more overdispersed across their phylogenies than those with higher diversification rates and that they tend to show distinct geographical distributions. We name this phenomenon *arrested diversification*, i.e. the evolutionary regime in which lineages show disproportionately low diversification, and discuss its implications for our understanding of the mechanisms that generate and maintain species diversity.

## Results

### Diversification rates

**Fig. 1:**
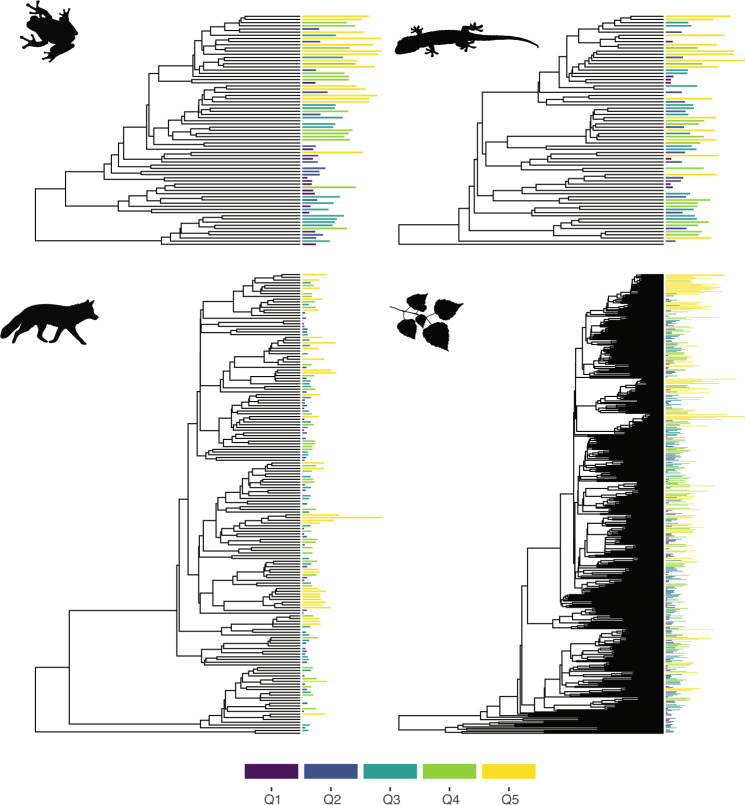
Phylogenetic distribution of diversification rates among amphibians, squamates, mammals, and flowering plants. Bars indicate the diversification rate of each family and were coloured according to five diversification quantiles. The colour palette was obtained using the viridis v0.5.1 package^50^.

**Fig. 2:**
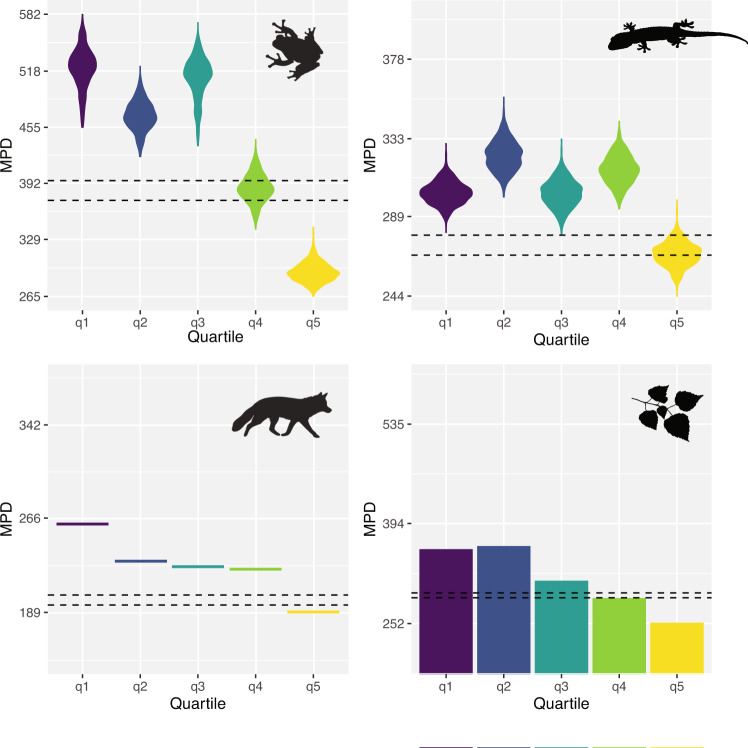
Mean pairwise distance of families in each of the quantiles indicated in Fig. 1 for amphibians, squamates, mammals, and flowering plants. The observed variations are due to differences in phylogenetic relationships and divergence times between topologies of each taxon. Confidence intervals based on resampling indicating expectations according to a random distribution across the tree are indicated as dashed lines. See text for details. The colour palette was obtained using the viridis v0.5.1 package^50^.

The phylogenetic distribution of families with different diversification rates, based on their corresponding quantiles, are shown in Fig. 1. Regardless of the studied taxon, there were differences among quantiles in their phylogenetic distribution (Fig. 1) that were confirmed by their comparison with null models of clustering/overdispersion. These analyses showed an overall trend of decreasing MPD with increasing diversification rates, even after accounting for phylogenetic uncertainty (*p* < 0.001, Fig. 2). When compared to the expectations based on resampling (dashed lines in Fig. 2), families in the lowest diversification quantiles are consistently more overdispersed than expected by chance, as opposed to the fifth quantile with the highest diversification rates. In other words, poorly-diversifying lineages tend to be more scattered throughout their phylogeny, whereas rapidly diversifying tended to be concentrated in particular regions of the tree. Finally, MPD values based on simulations were invariant across quantiles (Supplementary Fig. 1), reinforcing the interpretation that the geometry of the underlying trees is not sufficient to generate the phylogenetic distribution of diversification rates among quantiles.

### Spatial analyses

**Fig. 3:**
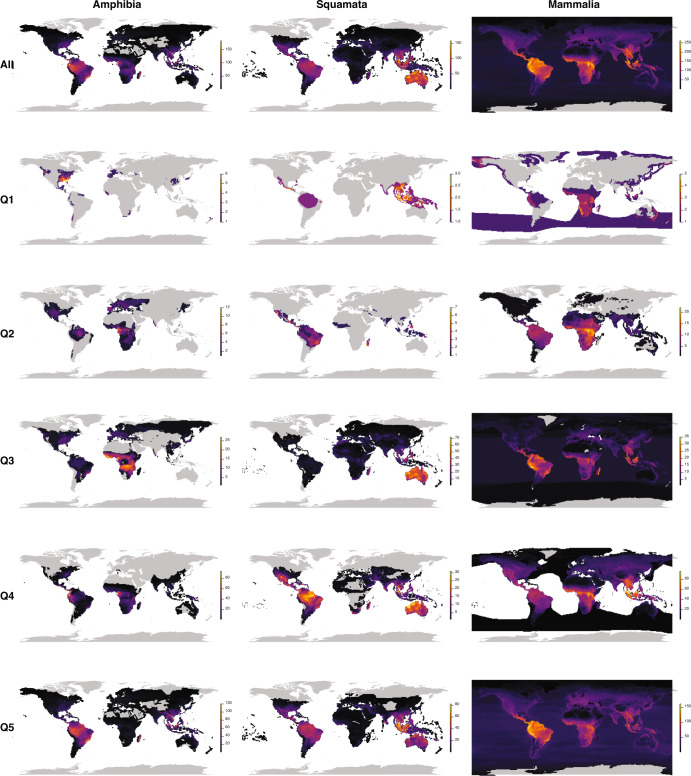
Species richness maps in each of the quantiles for amphibians, squamates, and mammals. All maps were constructed by the authors using the packages letsR v4.0^48^ and rgdal v1.5.32^51^. The colour palette was obtained using the viridis v0.5.1 package^50^.

Species richness maps showed substantial variation in spatial distributions between taxa across quantiles (Fig. 3). The first, third, and fourth quantiles differ considerably among mammals and squamates. There was some correspondence in the second quantile in the tropical and moist regions of Africa and South America. On the other hand, the fifth quantile was the most similar, with congruence areas in the Neotropics and Southeast Asia. By comparing different maps, it is possible to notice that the groups with the lowest diversification have low spatial correspondence with one another, with no evident pattern between them, whereas groups with higher diversification tend to be more concordant in their spatial patterns.

## Discussion

Particularly since the New Synthesis, when faced with the challenge of explaining variation in diversity across different branches of the tree of life, evolutionary biologists have traditionally focused on explaining the existence of highly diverse lineages (e.g.^17,18^). In this framework, normal levels of background diversification would occasionally be interrupted by events that would promote speciation. This focus on the “success stories” of the tree of life is reflected in the diversity of terms and concepts related to them, such as adaptive radiations^5,6^, key innovations^19^, the adaptive zones^6^, and ecological opportunity^20^. The results of the present study indicate that decreases in diversification rates might also play an important, but still poorly understood, role in explaining variation in diversity. The literature even lacks proper terminology to describe these phenomena, which might be one of the reasons why they are overlooked^21^. Some terms have been used to describe depauperate lineages, such as living fossils^22–24^, depauperons^14^, and relictual lineages^15^, but the actual mechanisms leading to poorly-diversifying lineages are largely unknown. Indeed, there is no comparable term to “adaptive radiation” that would apply to cases of negative diversification shifts. In this study, we propose a new term—arrested diversification—to describe the process by which lineages show disproportionately low diversification. Although one might argue that the distinction between normal diversification and arrested diversification might be arbitrary, so is the distinction between normal diversification and adaptive radiations, yet few would argue that such radiations exist in nature. Rather, we argue that those are points along a continuum^25,26^, and the introduction of this new term might spur efforts to understand why lineages are found on either end of the spectrum. It is important to note that the phenomenon of arrested diversification as described here does not distinguish between a slowdown in the diversification rate or a constant low diversification rate, as the estimator used by us does not allow for this differentiation.

The overdispersed phylogenetic distribution that was found in all analysed taxa in our study might provide some insight into the mechanisms that caused them to show arrested diversification. Indeed, our simulations suggest that this pattern is not simply a by-product of the geometry of phylogenetic trees. Rather, some lineages seem to have a state of low diversification and remain relatively species-poor over long periods of evolutionary time. Indeed, Barnes et al.^27^ found evidence that “dead clades walking”, clades that experienced a significant drop in diversity in mass extinctions events and were maintained with low richness until their extinction, are frequent in the fossil record and their origin is not restricted only to mass extinction events, which indicates that these clades may be a common component of many biotas. Interestingly, “dead clades walking” are distributed unevenly among phyla, with some lineages exhibiting a much higher proportion of these clades^27^.

One might be tempted to argue that arrested diversification is simply a consequence of a decrease in speciation rate or simultaneous decreases in speciation and extinction rates. For instance, Quental and Marshall^28^ suggested that failure to originate, what they called the “Entwives effect”, could have affected diversification patterns in Cenozoic terrestrial mammalian clades. However, as shown by Strathmann and Slatkin^29^, simple changes in time-homogeneous models, such as decreasing turnover rates while maintaining constant speciation and extinction rates or allowing for fluctuations in speciation and extinction rates are not sufficient to generate the observed patterns and might make it even more difficult for species-poor lineages to persist. Pie and Feitosa^15^ proposed two potential (non-exclusive mechanisms) to explain arrested diversification. First, species might become adapted to fairly stable, but specialized niches, which might essentially buffer them from extinction while at the same time preventing them from further diversification. Similar mechanisms have been proposed to explain the existence of “living fossils”, which would survive in refugia that would reduce the risk of predation^30–32^, but this is not common to all living fossils^33,34^. Second, lineages can remain in insular habitats, where the relatively depauperate local fauna and the reduced competition from other species might lead to long-term persistence. Although this seems to be the case in some lineages, such as *Sphenodon* in New Zealand^35^, the lack of congruence between the spatial distribution of lineages with arrested diversification is not consistent with this mechanism. In addition, species-poor phyla do not share similar habitats or life-histories^29^.

There are some limitations in our analyses that need to be kept in mind when interpreting our results. First, our estimates of diversification rates were calculated as the natural logarithm of the respective number of species divided by their corresponding stem age, and this method has been shown to be biased^36^ (see also^37^). In principle, we would prefer to use more powerful methods that are less affected by those biases, but that was not possible because of one or more of the following limitations: (1) they require fully-resolved phylogenies, whereas several of our trees are either family-level or involve considerable phylogenetic imputation in terminal nodes; or (2) are computationally-intensive and therefore would not be able to account for phylogenetic uncertainty in the case of phylogenies that are as large as those used in our study. However, we still believe that our results are robust, given that we used broad categories of diversification rates that do not require precise estimates of speciation and extinction rates. Indeed, the fact that we detected consistent patterns across such widely distinct taxa suggest that our conclusions are robust. Nevertheless, future studies might revisit these patterns as phylogenetic hypotheses become more robust and computational limitations are mitigated. It is important to note that, even though we mapped the distribution of species according to their diversification quantiles, we did not explicitly test for variation in potential correlates, such as productivity or climatic stability. We chose not to test them because our understanding of the evolution of poorly-diversifying lineages is still not sufficient to provide reasonable hypotheses to be tested. Moreover, if the mechanisms underlying the evolution of poorly-diversifying lineages involved those types of variables that are change consistently across geographical space, one would see concordant geographical distributions, which does not seem to be the case in the taxa investigated here (Fig. 3).

In conclusion, although considerable advances have been obtained in our understanding of the mechanisms that spur diversification (e.g.^5,8^), our understanding of the mechanisms that lead to arrested diversification are largely unknown. For instance, is arrested diversification reversible, or is it an evolutionary dead-end? Are there particular ecological conditions that favour arrested diversification? Is arrested diversification a cause or a consequence of limited phenotypic evolution? We hope that our study will stimulate new hypotheses and more rigorous tests of alternative mechanisms driving arrested diversification, and its relative contribution to the asymmetries in species richness found throughout the tree of life.

## Methods

### Data sources

We compiled data on phylogenetic relationships and species richness for a broad sample of organisms, namely seed plants, amphibians, squamates, and mammals. These taxa were chosen to ensure that our conclusions would be general across different ecologies and evolutionary histories. We focused on the taxonomic level of families as a reasonable trade-off between the description of variation in diversification rates while retaining relatively well-resolved phylogenetic resolution. Plant phylogenetic relationships and species richness data were based on Qian and Zhang^38^, whereas family species richness was obtained from The Plant List^39^. Amphibian, squamate, and mammal phylogenetic relationships and species richness data were based on Jetz and Pyron^40^, Tonini et al.^41^, and Faurby et al.^42^, respectively. In all taxa except for seed plants, which were represented by a single tree, phylogenetic relationships were obtained as a post-burnin sample of 1000 alternative topologies. Each tree was pruned, leaving only one representative for each family. The spatial distributions for mammals, squamates, and amphibians were retrieved from the IUCN Red List online database^43^.

### Diversification rates

Diversification rates of families of each taxon were calculated as the natural logarithm of the respective number of species divided by their corresponding stem age^44,45^. Except for plants, we used the median ages of the families for later calculations, instead of using mean ages, because the distribution of node ages among trees was highly skewed. Based on these results, we split each set of diversification rates of the taxa into five quantiles, with the first and fifth quantiles having families with the lowest and highest diversification rates, respectively. Then, we calculated the mean pairwise distance (MPD) of each quantile to assess whether they showed any signs of phylogenetic clustering or overdispersion using the ‘mpd’ function in picante v1.8^46^. We used this metric as it is straightforward to interpret, and because preliminary tests using different metrics provided qualitatively similar results. To account for phylogenetic uncertainty, we repeated MPD calculations across 1000 alternative topologies. To assess whether the observed MPD values are as expected given the studied topologies, the observed mean pairwise distance values were compared with a null model. This null model was constructed by attributing random families to the quantiles and calculating the MPD of the quantiles again. This procedure was repeated 1000 times and a 95% confidence interval was constructed from these estimates.

The choice of what constitutes a family is rather arbitrary and varies among taxonomic traditions and study organisms. To assess this potential bias, we simulated 1000 trees with 10,000 species using a pure-birth process with the ‘pbtree’ function in phytools v0.7-70^47^ as an additional null model. We considered all branches present at the beginning of the last fifth of the age of the entire clade as giving rise to families, and the number of species that accumulated on them after that as their correspondent richness using the ‘treeSlice’ function in phytools (Supplementary Fig. 2). We chose that cut-off value given that it approximates the ratio of family age to the age of the entire tree in our empirical datasets. We rescaled the simulated trees four times before calculating the mean pairwise distances to correspond to the total ages of the clades with the median age being used, except for plants. In this approach, we also constructed a 95% confidence interval for the mean pairwise distance values.

### Spatial analyses

Finally, we qualitatively assessed the level of spatial congruence among taxa in different quantiles by building their richness maps. Given limitations in data availability, these maps were not possible for flowering plants. We generated presence-absence matrices for families in each quantile using a global grid at a 1° resolution with the ‘lets.presab’ function in letsR v4.0^48^. All analyses were carried out in R 3.6.3^49^.

### Supplementary information

Suplementary Information

## Data Availability

All data used in this study was obtained from published sources. Family plant species richness were retrieved manually of the public database The Plant List^39^. Phylogenetic relationships were obtained from the papers Qian and Zhang^38^, Jetz and Pyron^40^, Tonini et al.^41^, and Faurby et al.^42^ for seed plants, amphibians, squamates, and mammals, respectively, checking the respective repositories cited in each of them. Spatial data was downloaded directly from the IUCN Red List online database^43^. None of these databases require previous permission nor include restrictions to access.
