# Supplementary material for: Arrested diversification? The phylogenetic distribution of poorly-diversifying lineages
Source: NPJ Biodivers. 2022 Dec 22;1:5. doi: 10.1038/s44185-022-00004-0 (PMC11290602; doi:10.1038/s44185-022-00004-0)
Supplement: Supplementary file 1 — Suplementary Information [file 44185_2022_4_MOESM1_ESM.pdf]

Suplementary Information

Arrested diversification? The phylogenetic distribution of poorly-diversifying lineages

Fernanda S. Caron[1] & Marcio R. Pie[2]

[1] Departamento de Zoologia, Universidade Federal do Paraná, C.P 19020, Curitiba PR 81531-990, Brazil.

[2] Biology Department, Edge Hill University, Ormskirk, Lancashire, United Kingdom.

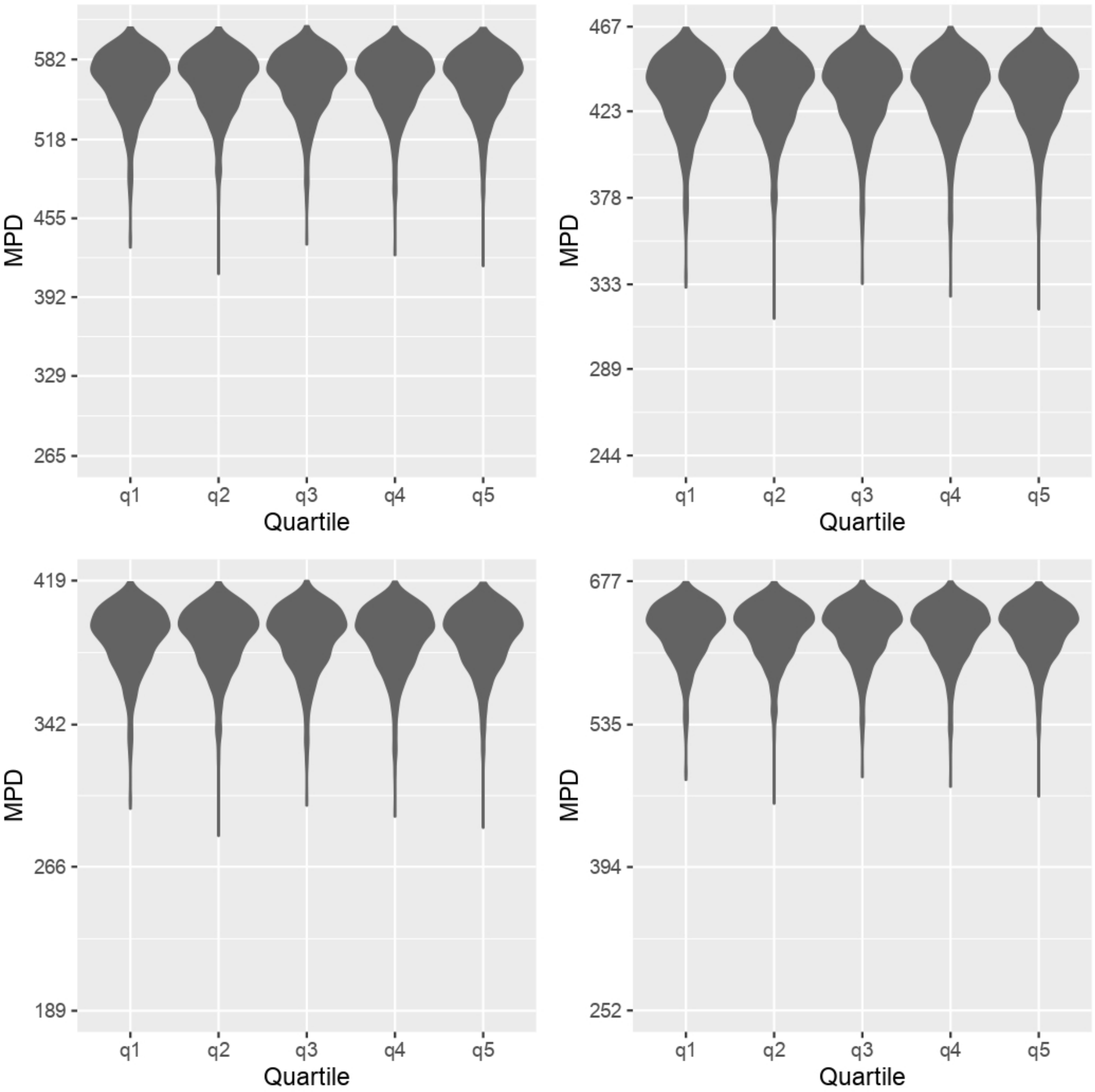

Supplementary Figure 1. Expected mean pairwise distance of the quantiles based on simulations for amphibians, squamates, mammals, and seed plants. See text for details.

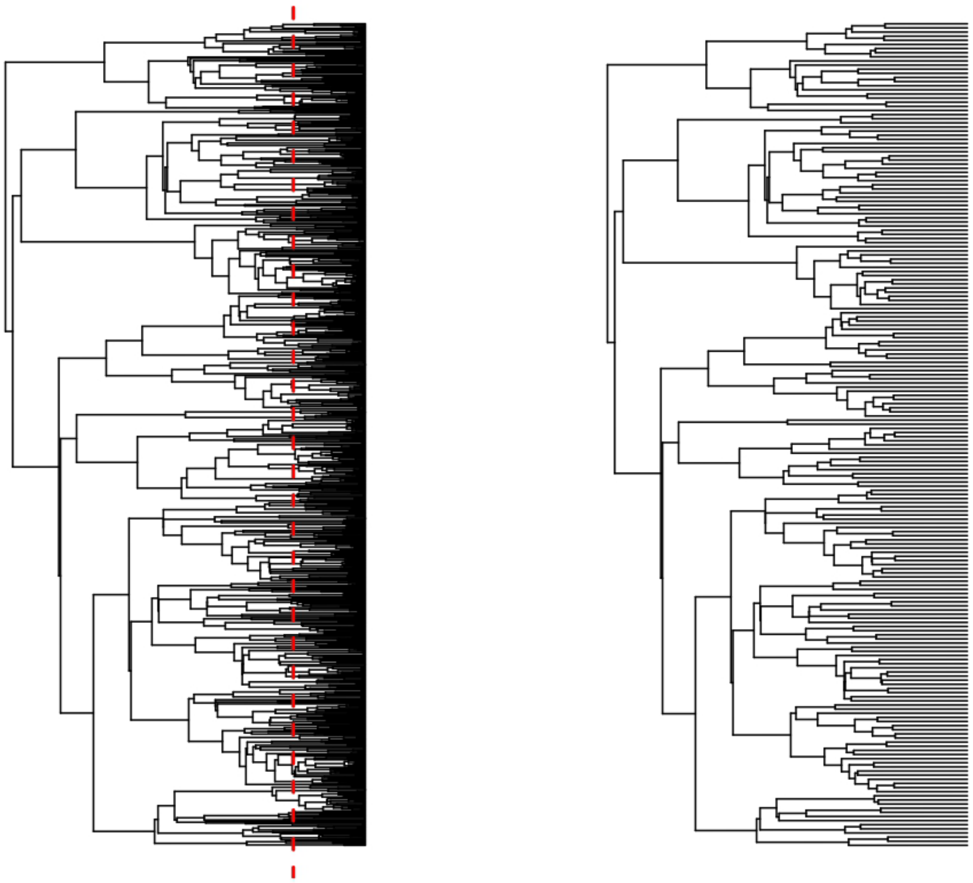

Supplementary Figure 2. Steps involved in simulations to account for taxonomic bias in the assessment of the phylogenetic distribution of diversification rates. Trees were simulated according to a pure-birth (Yule) process (left). All clades after the cut-off (red dotted line) were pruned so as to keep only one representative (right) and its clade richness was calculated. Finally, the distribution of diversification rates was then calculated as in the empirical datasets.
